# Newborn Screening for Congenital Infectious Diseases

**DOI:** 10.3201/eid1006.030830

**Published:** 2004-06

**Authors:** Eurico Camargo Neto, Rosélia Rubin, Jaqueline Schulte, Roberto Giugliani

**Affiliations:** * Universidade Federal do Rio Grande do Sul, Porto Alegre, Brazil,; † Nobel RIE Laboratory, Porto Alegre, Brazil,; ‡ Neonatal Screening Center, Porto Alegre, Brazil;; § Hospital de Clínicas de Porto Alegre, Porto Alegre, Brazil

**Keywords:** Neonatal screening, congenital infection, filter paper blood spots

## Abstract

To estimate the prevalence of congenital toxoplasmosis, Chagas disease, cytomegalovirus, and rubella, blood samples on dried blood spot (DBS) from neonates (day 3–20 of life) were screened for immunoglobulin (Ig) M against *Toxoplasma gondii*, cytomegalovirus, rubella virus, and IgG against *Trypanosoma cruzi* by methods used for serum and adapted for use with DBS. Positive samples were further analyzed for IgM and IgG in serum from neonates and mothers. DBS samples from 364,130 neonates were tested for *Toxoplasma gondii*–specific IgM, and 15,873 neonates were also tested for IgM against cytomegalovirus and rubella virus and for *Trypanosoma cruzi*–specific IgG. A total of 195 were diagnosed with congenital toxoplasmosis, 16 with cytomegalovirus, and 11 with congenital rubella. One newborn had a confirmed result for Chagas disease, and 21 mothers had positive serum antibodies. These results suggest that infectious diseases should be considered for future inclusion in programs for newborn screening of metabolic diseases in disease-endemic areas.

Toxoplasmosis infection during pregnancy can cause congenital infection and manifestations, such as mental retardation and blindness (*1*). Hydrocephalus, intracranial calcification, and retinochoroiditis are the most common manifestations of tissue damage from congenital toxoplasmosis. However, the effect of prenatal treatment on these outcomes is unclear (*2*), and the best method for preventing and controlling congenital toxoplasmosis is controversial. A neonatal screening program based on detecting immunoglobulin (Ig) M antibodies against *Toxoplasma gondii* alone would identify 70%–80% of congenital toxoplasmosis cases (*3*). Moreover, prenatal screening has indicated neither the natural history of toxoplasmosis nor the efficacy of antiparasite treatment during pregnancy (*4*). A study by Guerina et al. (*5*) showed a prevalence of congenital toxoplasmosis of 1 per 10,000 live births in the United States, where 85% of women of childbearing age are susceptible to acute infection with *T. gondii* (*6*).

Congenital Chagas disease has been reported, mostly in Latin America (*7*), where approximately 20 million persons are affected; 90 million others are at risk of being infected by the parasite (*8*). The high prevalence of the disease has been demonstrated in several Latin American countries (*8**–**10*). The evolution of the congenital and reactive forms of the disease has yet to be determined (*11*). The vertical transmission of *Trypanosoma cruzi* cannot be prevented, but early detection and treatment of congenital infection achieve cure rates close to 100% (*12**–**14*). Persons infected by *T. cruzi* can be successfully treated with nifurtimox or benzonidazole (*12**,**14*).

Cytomegalovirus is the most common congenital virus infection in the world. Both primary and recurrent infection can result in fetal infection. The birth prevalence of congenital cytomegalovirus infection varies from 0.3% to 2.4%, and at least 90% of congenitally infected infants have no clinical signs (*15*). The disease causes illnesses ranging from no clinical signs to prematurity, encephalitis, deafness, hematologic disorders, and death (*16*). Congenital cytomegalovirus infection is described in 30,000 to 40,000 newborns each year in the United States; approximately 9,000 of these children have permanent neurologic sequelae (*17*). The death rate of symptomatic congenital cytomegalovirus infection is approximately 30% (*18*). The value of vaccination against congenital cytomegalovirus infection is not known, and screening of newborn infants has been recommended to indicate infants at high risk for deafness and to make early rehabilitation possible (*18*).

Rubella virus infection during early pregnancy can lead to severe birth defects known as congenital rubella syndrome (*19*). Sequelae of rubella virus infection include three distinct neurologic syndromes: postinfectious encephalitis after acute infection, a range of neurologic manifestations after congenital infection, and an extremely rare neurodegenerative disorder, progressive rubella panencephalitis, that can follow either congenital or postnatal infection (*19*). A review of the literature that identified studies about the prevalence of anti-rubella antibodies from developing countries concluded that congenital rubella syndrome is an under-recognized public health problem and that appropriate data need to be collected to estimate the cost-effectiveness of a potential global rubella control program (*20*).

## Material and Methods

### Samples

Blood was obtained by heel puncture and applied on filter paper Schleicher and Schuell 903 (Keene, NH, USA), between day 3 and day 20 of life (mean day 10). The samples were collected in areas throughout Brazil and sent by mail to Porto Alegre, South Brazil, where the tests were performed.

### Tests in Filter Paper Dried Blood Spots (DBS)

An indirect enzymatic immunoassay for IgM anti–*Toxoplasma gondii* prepared in-house (*21*) was used to test the first 78,350 samples. A fluorometric capture enzymatic immunoassay (FEIA) (Neonatal *Toxoplasma gondii*, AniLabsystems, Helsinki, Finland) was used in 285,780 samples. A kit produced by Wiener Laboratory (Rosario, Argentina) to detect IgG against *Trypanosoma cruzi* in human serum was adapted. In brief, a 3.2-mm DBS paper disk was placed in microtiter plates precoated with *T. cruzi*. The serum was eluted with 200 µL of phosphate-buffered saline (PBS)/bovine serum albumin (BSA) buffer on an orbital shaker set at 100 rpm for 60 min and incubated for 14–16 h at room temperature. After washing with PBS/BSA buffer, the protocol was followed according to the manufacturer's instructions with two modifications: the reactions occurred at room temperature, and the incubation times were duplicated, except after the addition of the color reagent. Also, two kits produced by Diesse Diagnostica Senese (Monteriggioni, Italy) were adapted to detect IgM against cytomegalovirus and IgM against rubella in human serum eluted from filter paper. A 3.2-mm DBS paper disk was placed in microtiter plates precoated with an anti-human IgM monoclonal antibody. The serum was eluted with 150 µL of PBS/BSA buffer on an orbital shaker set at 100 rpm for 2 h at room temperature. Afterwards, the protocol was followed according to the manufacturer's instructions with the same modifications made with the IgG Chagas test.

### Controls

The cutoff for each test was obtained by testing 97 whole blood samples negative for IgM *Toxoplasma gondii* antibodies, 95 whole blood samples negative for IgG *Trypanosoma cruzi* antibodies, and 86 whole blood samples negative for IgM cytomegalovirus and rubella virus antibodies. The cutoff was established as three times the mean optical density of the negative samples. Negative, cutoff, and positive control samples were prepared in DBS for each test. The sensitivity of the methods was tested with 55 positive IgM *Toxoplasma gondii* samples, 43 IgG positive *Trypanosoma* samples, and 40 positive IgM cytomegalovirus and rubella samples. All samples were over the cutoff point for a preliminary analytical sensitivity of 100%. The presumptive positive samples were confirmed in a new duplicate run.

### Confirmatory Serologic Tests

Serum tests were performed on samples from the mothers and neonates. For the first 202 case-patients with possible congenital toxoplasmosis and Chagas disease, an indirect immufluorescence test (Biolab-Meriéux Diagnóstica, Rio de Janeiro, Brazil) was used. Confirmatory serum tests for toxoplasmosis, cytomegalovirus, and rubella (IgM and IgG) were run by microparticle enzyme immunoassay (MEIA) in the Axsym (Abbott Laboratories, Chicago, IL). The FEIA method was used for serum tests and run in parallel with the Axsym, which showed good agreement.

### Clinical Examination of Infected Infants

Patients suspected to have congenital toxoplasmosis and cytomegalovirus were given a skull ultrasound, tomography, or x-ray and ophthalmoscopic and audiologic exams. Patients suspected to have congenital Chagas disease and their mothers were evaluated for cardiac and esophageal malformations. Patients suspected to have congenital rubella were evaluated for hearing loss and eye lesions. When the samples were above or maximally 20% below the cutoff value, serum samples from the infant and the mother were requested. All clinical and follow-up information was obtained by contacting the pediatricians or, in rare cases, the families.

A neonate was followed and classified as infected by meeting one of the following criteria: antigen-specific IgM and IgG in the neonate and in the mother, antigen-specific IgM in the neonate only, antigen-specific IgM in the mother only, or increased amount of antigen-specific IgG in the neonate. An increase in the neonate's IgG antibodies excluded maternal origin.

## Results

### Congenital Toxoplasmosis

We analyzed 364,130 DBS samples for IgM against *Toxoplasma gondii*, and 699 samples were positive; all were recalled for serum confirmation. Serum samples from 594 neonates and 576 mothers were received, and of these, 202 suspected cases were tested by indirect immunofluorescence (IIF) (17 diagnosed with congenital toxoplasmosis), and 497 were tested by MEIA and FEIA (178 were diagnosed with congenital toxoplasmosis). A total of 195 neonates (1 in 1,867) were confirmed to have congenital toxoplasmosis. The laboratory findings are presented in Table 1, and the clinical findings are summarized in Table 2. The false-positive percentage was 0.16%. All patients with confirmed diagnoses were given sulphadiazine, pyrimethamine, and folinic acid.

**Table 1:** Serum results in the confirmatory tests

Disease	IgM^a^ in mother and neonate	IgM in neonate	IgM in mother	Increase of IgG in neonate
Congenital toxoplasmosis	84	21	49	41
Chagas disease	1^b^	1	1	1^b^
Cytomegalovirus infection	6	9 (2 adopted)	1^b^	1^b^
Congenital rubella	8 (4 mothers vaccinated)	9 (2 adopted)	2^b^	1^b^

^a^Ig, immunoglobulin. ^b^Same neonate.

**Table 2:** Symptoms and findings in patients with congenital toxoplasmosis

n	Complementary examination	Clinical findings
25	Retinal scar or retinochoroiditis	2 blind, 1 with myopia
14	Intracranial calcifications	1 with cognitive deficiency, 4 with splenomegaly or hepatosplenomegaly (HSM)
7	Retinal scar or retinochoroiditis and intracranial calcifications	2 with splenomegaly or HSM, 2 with neuromotor retardation, 1 microcephaly, 1 hydrocephaly and microophtalmya, 1 died immunosuppressed
7	Other symptoms	Splenomegaly or HSM, neuromotor retardation, microcephaly, hydrocephaly and microophthlmya, 1 died immunosuppressed

Of the 195 patients with congenital toxoplasmosis, 138 (70.7%) were asymptomatic until 7 years of age. One IgM-positive asymptomatic infant also had HIV, and six patients with sequelae received late treatment (6–14 months after diagnosis) and could be asymptomatic if treated early. The follow-up was 1–84 months (mean 30 months).

### Congenital Chagas Disease

We analyzed 15,873 DBS samples for Chagas disease and had 36 positive results. Serum samples from 31 neonates and 30 mothers were received for confirmatory tests. Results are shown in Table 1. The prevalence of specific *Trypanosoma cruzi*–specific IgG was estimated in 1 in 756 mothers (false-positive rate of 0.08%). All mothers and neonates had x-rays and echocardiographys. One mother (age 41) had an expanded heart and had a brother with Chagas disease. All others were asymptomatic and are under clinical observation. The antibodies observed in the neonates disappeared in time. The follow-up for positive children was 1–24 months (mean 15 months).

### Congenital Cytomegalovirus

A total of 15,873 DBS samples for IgM against cytomegalovirus were analyzed, and 39 were positive. Thirty-two serum samples from neonates and 30 from mothers were received for confirmatory testing, and 16 cases were confirmed (Table 1). In 8 case-patients, the IgG levels decreased in the neonates, and in 15 case-patients, IgG in the neonates' serum only was interpreted by the clinicians as being of maternal origin. Likewise, in one case, antigen-specific IgM was detected only in the mother's serum and antigen-specific IgG only in the neonate's serum. This neonate was not followed. The prevalence of cytomegalovirus was estimated at 1 in 992 live births, and the false-positive was 0.11%. The follow-up of the infected patients showed that 11 were asymptomatic until 2 years of age, including a premature baby (36 weeks' gestation). Laboratory results and clinical findings are presented in Tables 1 and 3. The follow-up was 1–24 months (mean 15 months).

**Table 3:** Pediatric decision and symptoms presented in patients with congenital cytomegalovirus

n	Decision	Symptoms
5	No treatment	Asymptomatic, clinical follow-up
6	Symptomatic; treated with ganciclovir	Microcephaly, intracranial calcifications, deafness, failure to thrive, HSM^a^ (initially investigated for galactosemia), progressive muscular atrophy, sepsis, low weight, difficulty swallowing (died), thrombocytopenia, leukopenia

^a^HSM, hepatosplenomegaly.

### Congenital Rubella

A total of 15,873 DBS samples for IgM against rubella virus were analyzed, and positive results were obtained in 67. Serum samples from 55 neonates and 52 mothers were received, and 16 were positive. Four mothers and one neonate were vaccinated against rubella and were excluded from the sample. The prevalence estimated was at 1 in 1,443, and the false-positive rate was 0.30%. Of 49 neonates and mothers without detectable IgM, 30 were followed until the specific IgG levels decreased. In 19 cases, the infant's IgG levels were interpreted by the pediatrician as being of maternal origin and were not followed. From these cases, three mothers received rubella vaccine before pregnancy. The follow-up of the 11 positive cases is shown in Table 4. The follow-up was 1–24 months (mean 15 months).

**Table 4:** Symptoms and number of patients with congenital rubella

Asymptomatic	Symptomatic
6 (1 adopted)	1 congenital rubella syndrome (died)
	2 with scars in 1 eye
	1 with cataract and received lens implant
	1 with cardiopathy and partial deafness

## Discussion

In screening for congenital toxoplasmosis, 195 neonates had diagnosis confirmed, and 105 (53.8%) had specific IgM. The diagnosis of 41 cases (21%) was only possible by monitoring the specific IgG levels in the infants; 49 (25%) cases were followed because of IgM in the mother's serum samples. Some mistakes may have occurred in the clinical evaluation of neonates in whom IgM were not detected in serum. A prospective study showed that a serologically transient toxoplasmosis occurred in 15% of the cases of unknown pathophysiology, leading to a risk of misdiagnosis and inadequate surveillance (*22*). The clinical decision not to monitor IgG levels in 123 patients for whom IgM was detected only in the mother's serum suggests that the concept still prevails: in the absence of IgM in the neonate, the IgG is from maternal origin. Several cases might have been misdiagnosed in these cases. In some cases, the beginning of treatment was delayed because of the following: 1) unwillingness of the clinician to treat asymptomatic infants because of the toxicity of the drugs; 2) time elapsed between birth, screening, confirmatory tests, and clinical examinations; and 3) the decision of the family to consult another physician.

Congenital toxoplasmosis is routine in prenatal studies in France, and the efficacy of this program is difficult to estimate, even considering the benefits (*23*). Moreover, prenatal programs have the risk of invasive methods and, according to Lebech (*3*), testing for specific IgM shows a better cost-benefit ratio if included in newborn-screening programs.

In 41 confirmatory serum tests for Chagas disease on samples from 21 neonates and 20 mothers, IgM antibodies were found in one neonate. He was treated and remains asymptomatic. IgG levels decreased in all asymptomatic neonates. One mother was identified with cardiac enlargement, and all the others received clinical counseling. In the population studied, most of the samples came from urban areas, and the incidence of mothers with specific antibodies (1 in 756) suggests that the seroprevalence can be higher in rural and disease-endemic areas (*9**,**11**,**12*). Because testing to detect IgM against *Trypanosoma cruzi* is not available, neonatal screening could detect asymptomatic mothers.

IgM against cytomegalovirus was detected in 87.5% of the patients diagnosed with congenital cytomegalovirus; 68.8% were asymptomatic. In 15 neonates, having only IgG antibodies in the serum was interpreted as being of maternal origin by the clinicians. The lack of information about the synthesis of specific antibodies against cytomegalovirus could be justified for the same reasons described previously for congenital toxoplasmosis (*22*). In a 16-year study, 388 children with congenital cytomegalovirus were evaluated for neurosensorial hearing loss (*24*). A hearing deficit was observed in 5.2% of the cases at birth and 15.4% in children >6 years of age, and neonatal screening for cytomegalovirus infection was suggested (*23*). Symptomatic cytomegalovirus can occur after maternal recurrent infection, but the incidence of these cases is still not established (*16*). Seropositive women reinfected by a different strain of cytomegalovirus can transmit the infection to the fetus and deliver a symptomatic child (*25**,**26*). In this work, the incidence of congenital infection by the cytomegalovirus was estimated to be 1 in 992. A successful treatment with the combined use of ganciclovir and anti-cytomegalovirus was reported (*27*).

Because of the mass vaccination to rubella, the high incidence of positive tests was unexpected (1 in 1,443, excluding the positive tests in vaccinated mothers). The results confirmed the findings of Cutts and Vynnycky (*20*) that the disease is under-recognized in developing countries. As observed with congenital toxoplasmosis and cytomegalovirus, 38.7% of neonates showed only IgG antibodies in the confirmatory tests. No further investigation was made because the clinicians presumed at follow-up that IgG was of maternal origin. Also, rubella vaccination of young women does not seem to be enough to prevent the transmission of the virus in a future pregnancy (*28*). However, prenatal care and mass vaccination seem to be the better choices to prevent new cases of congenital rubella. The purpose of neonatal screening would be to identify congenitally asymptomatic, infected neonates at birth. In Brazil (170 million persons and approximately 2,400,000 newborns/year), the prevalence of infectious diseases is higher than phenylketonuria (1 in 13,000) and congenital hypothyroidism (1 in 3,500). Congenital toxoplasmosis, with well-defined treatment protocols and a high prevalence, deserves special attention from health authorities, and its inclusion in screening programs should be considered. The follow-up of children until 7 years of age showed that most patients treated were asymptomatic or that the sequelae observed at the time of diagnosis had not progressed. By using the existing programs of newborn screening in the country, the inclusion of congenital toxoplasmosis, cytomegalovirus, and Chagas disease in disease-endemic areas would increase the cost of the program to approximately U.S.$1.50 per test. Also, treating infectious diseases is cheaper, and the time of treatment is shorter when compared to the expensive and long-term treatment of metabolic diseases. Studies on long-term follow-up of these children are in progress for a better understanding of the efficacy of the treatments and the effectiveness of mass screening.
